# Improving the Sensitivity of Fourier Transform Mass Spectrometer (Orbitrap) for Online Measurements of Atmospheric Vapors

**DOI:** 10.1021/acs.analchem.2c03403

**Published:** 2022-11-07

**Authors:** Runlong Cai, Wei Huang, Melissa Meder, Frederic Bourgain, Konstantin Aizikov, Matthieu Riva, Federico Bianchi, Mikael Ehn

**Affiliations:** † Institute for Atmospheric and Earth System Research/Physics, Faculty of Science, University of Helsinki, Helsinki 00014, Finland; ‡ Univ Lyon, Université Claude Bernard Lyon 1, CNRS, IRCELYON, Villeurbanne 69626, France; § Thermo Fisher Scientific (Bremen), Bremen 28199, Germany

## Abstract

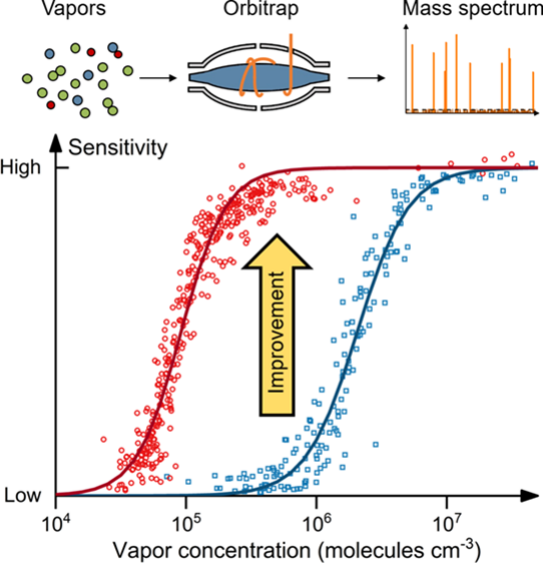

Orbitrap Fourier transform mass spectrometry coupled with chemical ionization (CI) is a new-generation technique for online analysis in atmospheric chemistry. The advantage of the high resolving power of the CI-Orbitrap has been compromised by its relatively low sensitivity to trace compounds (e.g., <10^6^ molecules cm^–3^) in complex gaseous mixtures, limiting its application in online atmospheric measurements. In this study, we improve the sensitivity of a Q Exactive Orbitrap by optimizing the parameters governing the signal-to-noise ratio. The influence of other parameters related to ion transmission and fragmentation is also discussed. Using gaseous compounds in an environmental chamber, we show that by increasing the number of ions in the analyzer, the number of microscans (i.e., transients), and the averaging time, the sensitivity of the CI-Orbitrap to trace compounds can be substantially improved, and the linear detection range can be extended by a factor of 50 compared to standard settings. The CI-Orbitrap with optimized parameters is then used to measure oxygenated organic molecules in the atmosphere. By improving the sensitivity, the number of detected compounds above the 50% sensitivity threshold (i.e., the signal intensity at which the sensitivity is decreased by half) is increased from 129 to 644 in the atmospheric measurements. The Q Exactive CI-Orbitrap with improved sensitivity can detect ions with concentrations down to ∼5 × 10^4^ molecules cm^–3^ (1 h averaging), and its 50% sensitivity threshold is now below 10^5^ molecules cm^–3^.

## Introduction

As major contributors to secondary pollutants such as secondary organic aerosols and tropospheric ozone, volatile organic compounds (VOCs) and their oxidation products play important roles in atmospheric physicochemical processes. Identifying these organic vapors is fundamental to the assessment of their impacts on human health, air quality, and the climate. However, via rapid oxidation, freshly emitted VOCs with the same composition can form numerous oxygenated organic molecules (OOMs) with volatility and concentrations spanning over a broad range.^1−3^ Measurements of atmospheric vapors such as OOMs require analytical techniques with high resolving powers to distinguish among different compositions, low limits of detection (LOD) to separate the signal of trace compounds from instrumental noise, and sufficient temporal resolution to follow the variation of vapor concentrations.

The online time-of-flight (ToF)-based mass spectrometry (MS) has been widely used to analyze OOMs in atmospheric environments and laboratory studies.^4^ Coupled with different ionization techniques, ToF-MS can measure gas-phase and particle-phase organic compounds in different volatility ranges.^4−6^ Averaging the millisecond-scale raw data of ToF-MS up to minutes yields good LOD for atmospheric measurements. For instance, with an atmospheric pressure chemical ionization (CI) technique for gas-phase vapor detection, the LOD of a nitrate CI-ToF-MS was reported to be 3.6 × 10^4^ molecules cm^–3^ for 15 min averaging.^7^ The resolving power of a ToF system can range from a few hundred up to 50,000, yet it rarely exceeds 15,000 for typical online atmospheric measurements.^8^ Multi-reflection ToF instruments can significantly increase the ion flight path, resulting in higher mass resolving powers.^9^ However, they are seldom applied in atmospheric measurements owing to the low sensitivity. The limited resolving power of online ToF-MS may cause interferences among ion signals, which unavoidably introduce uncertainties to the identification of numerous vapors in complex atmospheric conditions,^10,11^ especially for polluted environments with various nitrogen-containing OOMs.^12,13^

Replacing conventional online ToF-MS with high-resolution Orbitrap MS can greatly improve atmospheric vapor identification. The mass resolving power of a Q Exactive Orbitrap^14^ MS at a mass-to-charge ratio (*m*/*Q*) of 200 Th can be in excess of 100,000. This greatly reduces the interferences among isobaric compounds and facilitates the unambiguous assignment of measured peaks to compounds compared to conventional online ToF-MS.^8^ Recent studies have shown that Orbitrap MS (referred to as Orbitrap below) coupled with different ionization techniques for atmospheric chemistry^15−17^ is a promising tool for online analysis of gas-phase and particle-phase OOMs.

However, the detection of trace OOMs poses a significant challenge to the mass spectrometric approaches as the OOM concentrations are often too low to produce signals with sufficient signal-to-noise ratios (SNRs), or even to go over the LOD. At elevated noise levels, all the reported values tend to deteriorate (e.g., mass accuracy, signal intensity, etc.). For the Orbitrap, signal intensities below a certain threshold need an intensity-based correction due to underestimations in inferred concentrations.^15^ While such a correction does improve the fidelity of the approach, it has no effect on the LOD, and does not address the uncertainties in the obtained results due to the corruption of the mass spectra by noise.

As the SNR levels dictate the overall sensitivity of the approach, the most straightforward way to improve the sensitivity is to increase the number of ions in the Orbitrap analyzer during the detection event by raising the automatic gain control (AGC) target,^18^ and consequently the signal. This works well if the amount of analyte is sufficient; for less abundant species, especially in highly heterogeneous samples, it may pose a challenge as the ion optics including the C-trap and the Orbitrap can contain only a finite number of ions.^15^ Therefore, alternative strategy based on signal averaging^19−24^ along with the optimization of other parameters is explored herein to improve the sensitivity of the CI-Orbitrap to detect atmospheric OOMs.

In this study, we aim to optimize the sensitivity of a CI-Orbitrap (Q Exactive Plus). First, we sample gas-phase OOMs from chamber experiments to investigate different governing parameters, e.g., the number of ions in the Orbitrap analyzer via adjusting the AGC target and the number of microscans for signal averaging. Other parameters influencing the sensitivity and the LOD of the spectra such as spectral averaging, the temperature of the inlet capillary, radio frequency (RF) amplitude of the stacked-ring ion guide (S-lens), and the measured mass range, are also investigated. After optimizing these parameters, we use the CI-Orbitrap to measure OOMs in the urban atmosphere of Helsinki, Finland. Based on these investigations, we give recommendations on the operations of CI-Orbitrap in measuring trace vapors.

## Experiments

**Figure 1:**
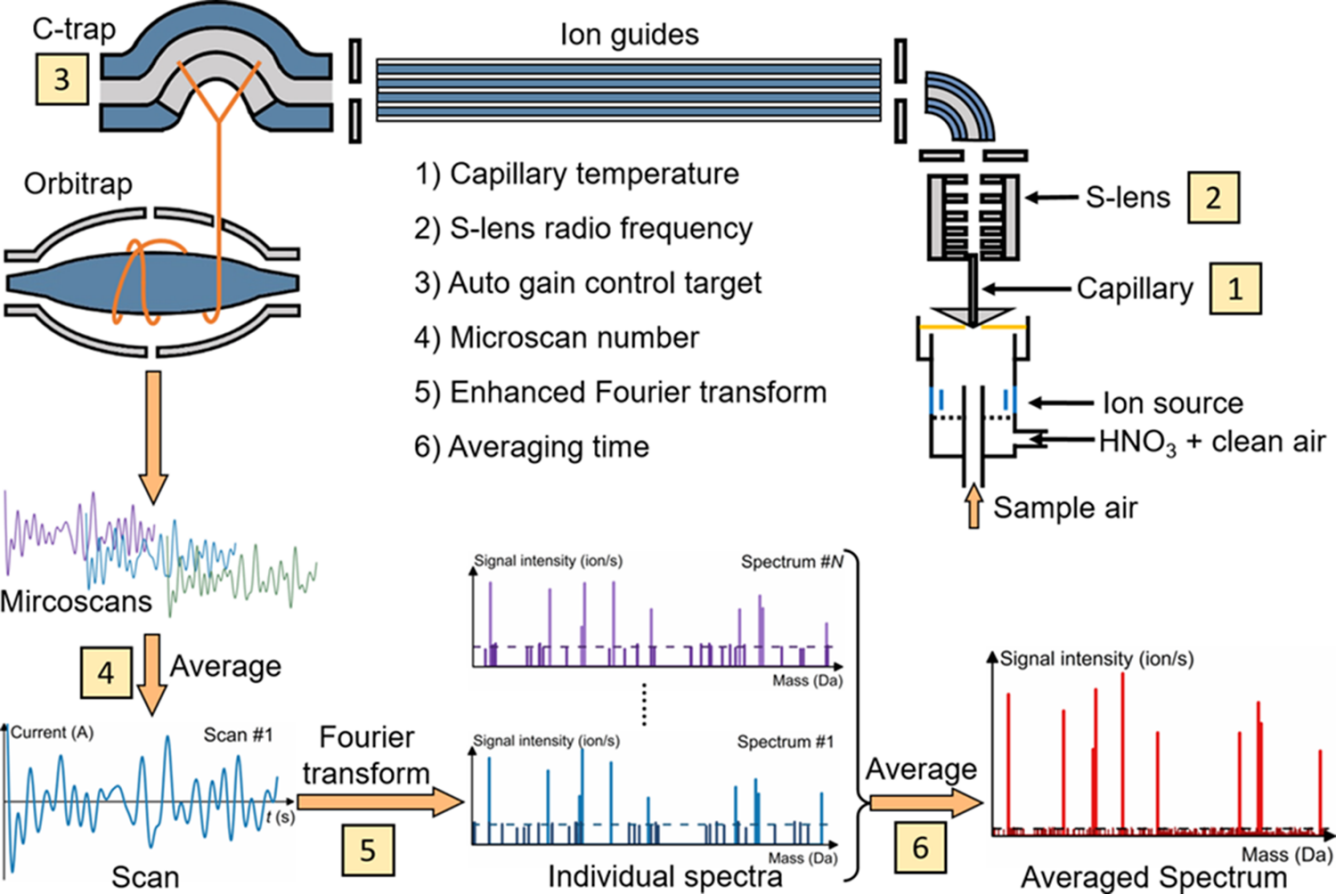
Illustrative schematic of the chemical ionization Orbitrap Fourier transform mass spectrometry. The influencing parameters investigated in this study are indicated by the numbers. The data reported by the Orbitrap are the mass spectra corresponding to every scan. Converting a scan into a mass spectrum in step 5 may reduce the spectral sensitivity. A series of individual mass spectra can be further averaged (in step 6) for further analysis. The noise peaks in a spectrum are the outliers of noise and their average height is indicated by the horizontal dashed line in the spectrum.

The data reported in this study were acquired with a research-grade Q Exactive Plus Orbitrap^14,25^ MS (Thermo Fisher Scientific Inc.). The mass resolution setting of the Orbitrap at *m*/*Q* = 200 Th was 280,000. The sample air containing trace vapors such as OOMs entered an Eisele-type CI-inlet.^26^ Neutral gas-phase OOMs were charged in the inlet using NO_3_^–^ and HNO_3_·NO_3_^–^ as reagent ions, which were generated from gas-phase HNO_3_ using a soft X-ray ion source (Figure 1). The contribution of larger reagent ions, e.g., (HNO_3_)_2_·NO_3_^–^, to the total intensity of reagent ions were negligible. The Orbitrap was operated in negative ion mode. The sampling flow rate (15 L min^–1^), sheath flow rate (30 L min^–1^), and voltages for the CI-inlet (−144 and – 131 V) were optimized to improve the signal and then kept constant during the experiments.

We investigated the sensitivity of the CI-Orbitrap to the measured OOMs and its influencing parameters with chamber experiments. The volume of the chamber was 2 m^3^ and the total flow rate of clean air entering the chamber was 40 L min^–1^. This chamber had been used to study the oxidation of VOCs (e.g., monoterpenes) and their contributions to secondary organic aerosol particles.^27,28^ Hence, OOMs composed of C, H, O, and possibly N remained as background residues (∼5 × 10^8^ cm^–3^) in the chamber. Their low and stable concentrations were optimal for testing the CI-Orbitrap sensitivity. To further minimize the influence of potential temporal variations of OOM concentrations during the experiments, the sensitivity as a function of each influencing parameter was determined twice during the increase (upscan) and the decrease (downscan) of the varying parameter.

After improving the sensitivity of the CI-Orbitrap, we tested its performance in atmospheric measurements in June–July 2021. The sampling site is located on the fourth floor of the Physicum building on the Kumpula campus of the University of Helsinki, Finland. A road with a bus stop is right below the sampling inlet (on the ground level). The nearest main road was ∼100 m away. The ambient air was sampled through a 1.2 m long 3/4 inch Teflon tube. The parameters for the CI-Orbitrap were switched every half an hour between two parameter settings when they were tested in atmospheric measurements.

The raw mass spectra reported by the CI-Orbitrap were analyzed using Orbitool^13^ (version 2.1.3, last access date November 8, 2021), which was designed for analysis of long-term online atmospheric data sets measured by Orbitrap MS. To quantify the intensity of noise in the reported spectra, we first selected all the peaks in a mass defect range of [−0.5, −0.2] Th, as there were no real ions there. All peaks with intensities below the 90th percentile within this mass range were then used to calculate a mean (μ) and standard deviation (σ). The mean noise intensity and the LOD were determined as μ and μ + 3σ, respectively.

## Theory

Simplified working principles of the CI-Orbitrap are shown in Figure 1. After ionization, an OOM becomes either a NO_3_^–^ clustered ion or a deprotonated ion. Ions are directed into the Orbitrap MS through a heated capillary. They are then transferred to the C-trap via a series of ion guides (including the S-lens). The ions are accumulated in the C-trap until a certain AGC target is reached. Then the accumulated ions are injected simultaneously into the Orbitrap analyzer. The signal obtained from these ions is referred to as a “microscan” or transient. Multiple microscans are averaged into a full scan, which is then converted into a mass spectrum via an enhanced Fourier transform (eFT) algorithm.^29^ Additional averaging of these mass spectra can further decrease the LOD. The concepts and parameters related to signal and noise are summarized in Table 1.

**Table 1:** Concepts and Parameters Related to Signal and Noise

limit of detection	the lowest distinguishable signal against noise. It is calculated as μ + 3σ, where μ and σ are the mean and standard deviation of the noise, respectively	
sensitivity	the increase of signal intensity per increase of sampled molecule concentration	SNR↑ ⇒ sensitivity↑
AGC target	the target number of ions in the Orbitrap analyzer	AGC target↑ ⇒ SNR↑
microscan	one complete mass analysis including ion accumulation and detection	microscan number↑ ⇒ SNR↑
scan	the average of microscans. One scan is converted into one individual spectrum *via* Fourier transform	
individual spectrum	one mass spectrum converted from on scan *via* Fourier transform	
averaged spectrum	averaging results of multiple individual spectra. Spectral averaging does not affect the sensitivity since it is performed after the Fourier transform	spectral averaging↑ ⇒ LOD↓

As the sensitivity of the CI-Orbitrap to the measured OOMs is determined by the SNR of OOM peaks in individual (i.e., unaveraged) spectra, it is worth briefly discussing the nature of the signal and the noise before launching into the subject of optimization. The signal, generated by ions oscillating in the Orbitrap analyzer, is concentrated around its resonant frequency. The noise, however, which is mostly thermal and electronic in nature,^30^ is spread throughout the entire frequency range and centered at zero intensity. Therefore, in addition to increasing the AGC target, the SNR can be boosted by increasing the number of observations, which leads to the different responses of the signal and the noise: the former tends to increase linearly whereas the latter as the square root of the number of observations. This results in the net increase of the SNR proportional to the square root of the number of observations.

One way to increase the number of observations is to extend the residence time of ions in the Orbitrap analyzer, which is known to boost not only the resolution but also the SNR.^31^ However, with an insufficient number of ions, the ion statistics will not improve significantly. Alternatively, averaging multiple microscans addresses the SNR issue as each microscan is an independent FTMS experiment. Microscan averaging has been known to be an efficient tool in the analysis, characterization, and sequencing of biological macromolecules and their complexes.^19,21−23^

Unlike the time domain data, FT mass spectra are strictly non-negative. Averaging FT mass spectra mainly affects the outliers above the noise threshold, which does not result in as strong of a de-noising effect as that on the microscan level.^18^ However, spectral averaging can improve the LOD of spectra and help distinguish signal from the noise outliers (referred to as noise peaks below), which can otherwise be difficult if the LOD is higher than certain signal peaks. As shown in step 6 in Figure 1, these noise peaks spread randomly and sparsely in each individual spectrum. Averaging individual spectra reduces their intensity and increases their number.

## Results and Discussion

We mainly focus on the governing parameters for the LOD and the SNR, namely the AGC target, the microscan number, and spectral averaging. Some other influencing parameters related to ion transmission, ion fragmentation, and overall spectral quality, i.e., the temperature of the inlet capillary, RF amplitude of the stacked-ring ion guide (S-lens), the measured mass range, as well the decision to use the eFT algorithm or reporting spectra in the magnitude mode, are addressed in the Supporting Information.

### AGC Target

**Figure 2:**
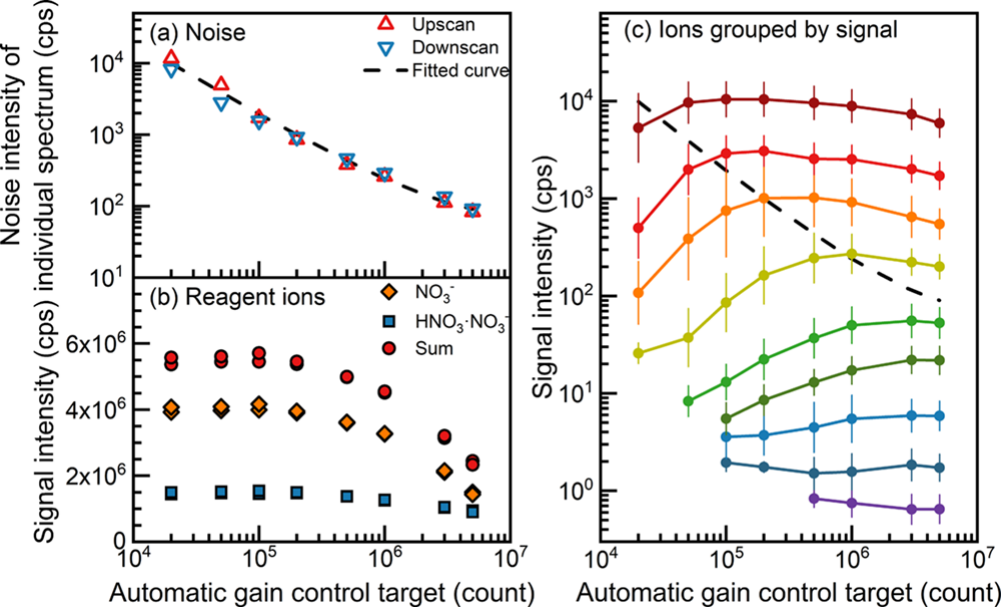
The influence of the automatic gain control target of ions on the measured (a) noise of individual spectra, (b) reagent ions, and (c) oxygenated organic molecules (average of upscan and downscan) during the chamber experiments. The automatic gain control target was gradually increased in the upscan and then decreased in the downscan. The microscan number was set to 1 in this test. The measured oxygenated organic molecules in (c) are grouped by their signal at automatic gain control target = 5 × 10^6^. The variation bar indicates the standard deviation of the ion signal in the same group. The dashed curve in (c) is identical to the fitted curve in (a). The signal is obtained by spectral averaging for 1 h.

As shown in Figure 2a, the mean noise of individual spectra decreases almost linearly with an increasing AGC target. The slope of the fitted curve is −1 on the log–log scale (after correcting ion losses at high AGC targets) because the SNR tends to be linearly proportional to the AGC target.

The dependence of the measured OOM signal on the AGC target is shown in Figure 2c. To characterize the influence of the AGC target at different OOM concentrations, we group peaks by their intensities (in counts per second, cps) measured at the highest AGC target (5 × 10^6^) and show the mean intensity of each group. With relatively constant OOM concentrations in the chamber, the measured peak intensities generally increase with an increasing AGC target. This increase is significant when the OOM signal is close to the noise level of individual spectra, i.e., the SNR for individual spectra is close to one. In contrast, the intensities of OOM peaks well above the noise (i.e., with a high SNR) are insensitive to the decrease of noise. The peaks well below the noise of individual spectra (as shown in Figure 2c) are obtained after further spectral averaging for 1 h, which decreases the noise of the resulting spectrum.

However, a high AGC target may cause some ion losses in the C-trap. We use the reagent ions (NO_3_^–^ and HNO_3_·NO_3_^–^), which are most abundant among the measured ions, to quantify the losses as the non-linearities in their sensitivities are negligible. As shown in Figure 2b, the peak intensities of the reagent ions are relatively constant for AGC targets below 10^5^ and then gradually decrease with an increasing AGC target. For the same reason, the OOM signal that is above the noise decreases when the AGC target is raised above 10^5^ (Figure 2c). We did not observe significant dependence of this decrease on the mass of OOM in these experiments.

Deciding on an optimal AGC target requires balancing the improvement of the sensitivity to low signals and the increased ion losses in the C-trap. The decision depends on the overall aim and the analytes, and will thus be study-specific. Here, we chose 10^6^.

### Microscan Number

**Figure 3:**
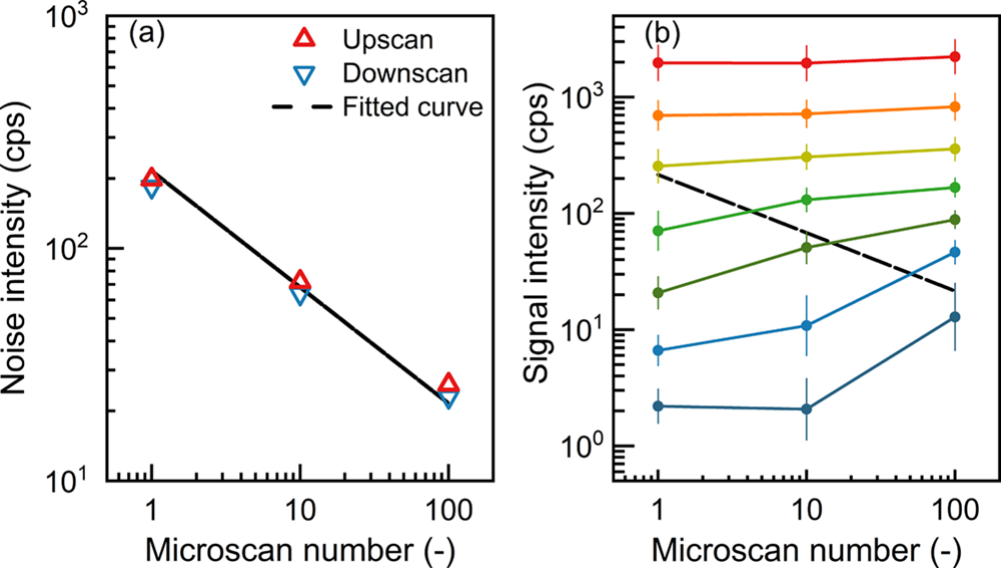
The influence of the number of averaged microscans on the measured (a) noise of individual spectra and (b) oxygenated organic molecule concentration during the chamber experiments (average of upscan and downscan). The automatic gain control target is 10^6^ for the results in this figure. The measured oxygenated organic molecules in (b) are grouped by their signal at microscan number = 1. The variation bar indicates the standard deviation of signal intensities in the same group. The dashed curve in (b) is identical to the fitted curve in (a). The signal is obtained by spectral averaging for 1 h.

Increasing the number of microscans further improves the SNR, as indicated by the correspondingly decreasing noise of individual spectra in Figure 3a. Since the SNR tends to increase proportionally to the square root of the number of observations, the slope of the fitted curve is −0.5 on the log–log scale. As a result, the measured signal of the trace OOMs increases with the increasing microscan number, which is especially pronounced for species with intensities close to the noise on the individual spectra level (Figure 3b). Compared to those low-intensity peaks, the fidelity of high-intensity peaks is not significantly improved by microscan averaging, as also indicated by the independence of reagent ion concentrations on the microscan number (see Figure S1 in the Supporting Information).

Increasing the AGC target and the microscan number improves the SNR albeit at the expense of the temporal resolution of the MS data. For the AGC target = 10^6^ and microscan number = 100, a scan during the chamber experiments (with a mass resolution setting of 280,000) takes ∼2 min, which meets the demand of most OOM measurements in atmospheric conditions.

### Spectral Averaging

Spectral averaging can further decrease the noise of the averaged spectra to facilitate the identification of the trace OOMs. For example, the data in Figures 2c and 3b are obtained after spectral averaging for 1 h so that the signal of the trace OOMs can be higher than the noise peaks of the averaged spectra. Similarly, the low signal with a low AGC target in the bottom left of Figure 2c is not shown because even after spectral averaging, it does not exceed the LOD. As shown in Figure S2, the height of noise peaks decreases inversely to the averaging time. The slope of the fitted curve is −1 on the log–log scale, which is consistent with the discussions in Theory.

Comparing the 8 h average LOD with AGC target = 10^6^ and microscan number = 100 to that of individual spectra with AGC target = 2 × 10^4^ and microscan number = 1, we show that spectral noise can be reduced by 5 orders of magnitude from ∼10^4^ to ∼10^–1^. Figure S2 also shows that the LOD of averaged spectra is governed by the temporal resolution of the averaged spectra and it is only weakly dependent on AGC target and the microscan number (as the number of averaged spectra has to change accordingly).

### Sensitivity

**Figure 4:**
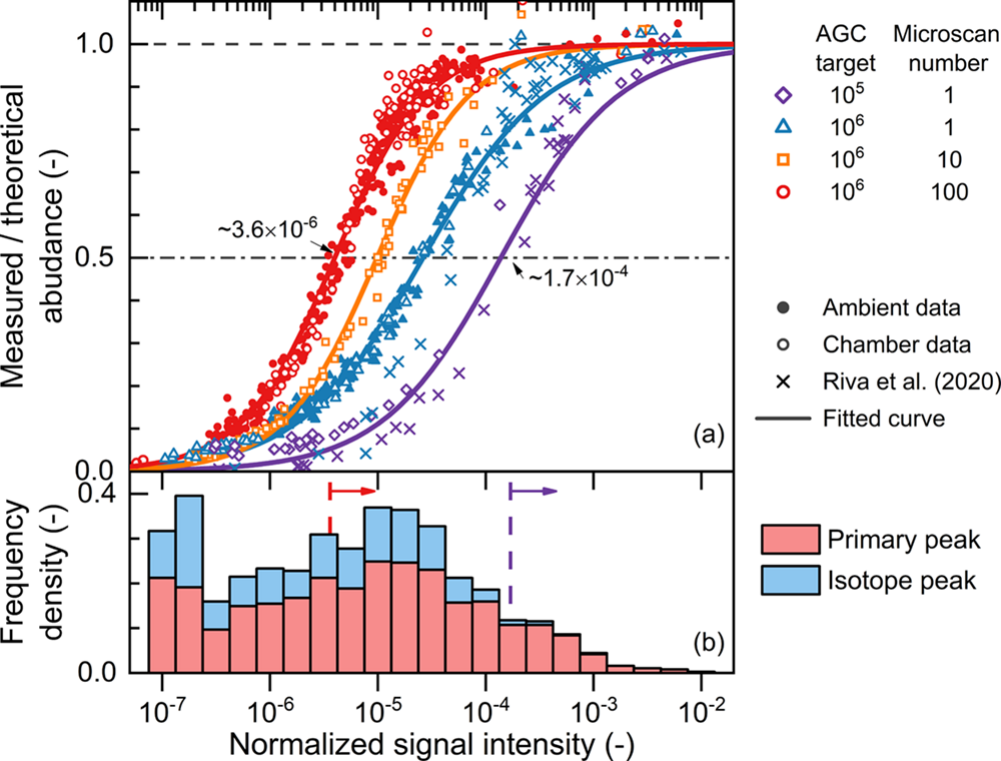
The Orbitrap sensitivity and the intensity distribution of measured signal of ambient gaseous compounds. (a) The sensitivity of the CI-Orbitrap as a function of signal intensity. The horizontal axis is the signal intensity normalized by dividing it by the total intensity of the reagent ions ([NO_3_^–^] + [HNO_3_·NO_3_^–^]). The vertical axis is the measured abundance of ions containing less abundant isotopes divided by the theoretical abundance calculated from the primary peak. Only the isotope peaks are shown in panel (a). Due to the low sensitivity of the Orbitrap at low signal, the ratio of measured abundance to theoretical abundance decreases with a decreasing signal. The markers indicate measured data and the curves indicate fits. The solid and open markers show the results from the atmospheric measurements and the chamber experiments, respectively. The crosses show the data from a previous report using a different nitrate CI-Orbitrap.^15^ (b) The distribution of average compound intensity measured in the atmosphere of the city of Helsinki (see Figure 5). The frequency gives the number of all unique peaks in the intensity range and it is characterized by the area (rather than height) of each bar. “Primary peak” refers to ions where each atom is the most abundant isotope (e.g., ^12^C, ^16^O, etc.), while all other ions are termed “isotope peaks.” The dashed lines and arrows indicate the 50% sensitivity thresholds for the two different settings.

To expand on the above discussions, we use the measured isotope abundances to indicate the sensitivity of the CI-Orbitrap. Molecules with less abundant isotopes (e.g., ^13^C) are expected to show a lower signal than the value predicted using natural abundances (e.g., ^13^C:^12^C = 1.12%) if sensitivity is a function of concentration. As shown in Figure 4a, the relative abundance of measured isotopic OOMs compared to theoretical isotopic abundance does indeed decrease with decreasing intensity.

In Figure 4, the measured intensities of isotopic OOMs are normalized by dividing them by the total intensities of the reagent ions ([NO_3_^–^] + [HNO_3_·NO_3_^–^]). We use normalized intensities instead of absolute intensities for three reasons. First, the normalization corrects signal variations caused by the fluctuation of the reagent ion concentrations. Second, the concentrations of OOM ions are proportional to the reagent ion concentrations in the CI-inlet; hence, the normalized signal can be readily converted to the measured concentration by multiplying it by a calibration factor.^32^

Third and most importantly, the total concentration of reagent ions affects the SNR of the CI-Orbitrap. At a certain AGC target, the number of OOM ions accumulated in the C-trap is governed by the ratio of the OOM concentration to the total ion concentration. Since the reagent ions are much more abundant than the OOM ions, the total concentration of reagent ions is almost equal to the total ion concentration. That is, increasing the reagent ion concentrations decreases the number of OOM ions accumulated in each microscan. As a result, the SNR is determined by the normalized signal rather than the absolute signal. Similarly, the ion losses in the C-trap at a high AGC target (see Figure 2) decreases the signal of both reagent ions and OOM ions with an insignificant mass dependence, hence the OOM concentrations calculated using the normalized signal and the calibration factor should be less affected than the absolute signal.

Increasing the AGC target and the microscan number improves the sensitivity of CI-Orbitrap for trace compounds, while spectral averaging further improves the identification of the signal peaks (Figure 4a). A 50% sensitivity threshold is herein taken as the normalized intensity at which the sensitivity is reduced by half (i.e., the measured abundance of an isotope is 50% of the theoretical abundance). Compared to a setting with AGC target = 10^5^ and microscan number = 1, increasing the AGC target to 10^6^ and the microscan number to 100 decreases the 50% sensitivity threshold from 1.7 × 10^–4^ to 3.6 × 10^–6^. Assuming a typical calibration factor of 1 × 10^10^ cm^–3^ for nitrate CI-inlets,^7,8,33^ the 50% sensitivity threshold of 3.6 × 10^–6^ corresponds to a concentration of 7.2 × 10^4^ cm^–3^ (after correcting the 50% sensitivity). The signals below the 50% sensitivity threshold can also be detected and corrected using the fitted sensitivity curve,^15^ given that they exceed the LOD of the averaged spectrum (see also Figure S4). For instance, the LOD with 1 h averaging is <5 × 10^4^ cm^–3^ with the estimated calibration factor. We note that, e.g., mass-dependent transmission and inlet losses may cause the calibration factor to reach 1 × 10^10^ cm^–3^ and above, resulting in an underestimation of OOM concentrations. Nevertheless, these are promising values for the measurements of ambient OOMs.

### Other Influencing Parameters

The influence of the eFT algorithm, capillary temperature, RF level of S-lens, and the mass-to-charge range of the spectrum on the measured signals was also explored. The eFT algorithm not only qualitatively increases the effective mass resolution of the Orbitrap mass spectra, but also filters out a significant number of noise peaks resulting in the overall improvement of the sensitivity (Figure S4). For the experiments in this study, we optimized the measured OOM signals with a capillary temperature of 150 °C, an S-lens RF amplitude of 40, and a mass-to-charge range of 50–750 Th (Figures S5–S7). Note that the optimal temperature and S-lens RF amplitude can be case-specific. More details on these results are given in the Supporting Information.

### Ambient Measurements

**Figure 5:**
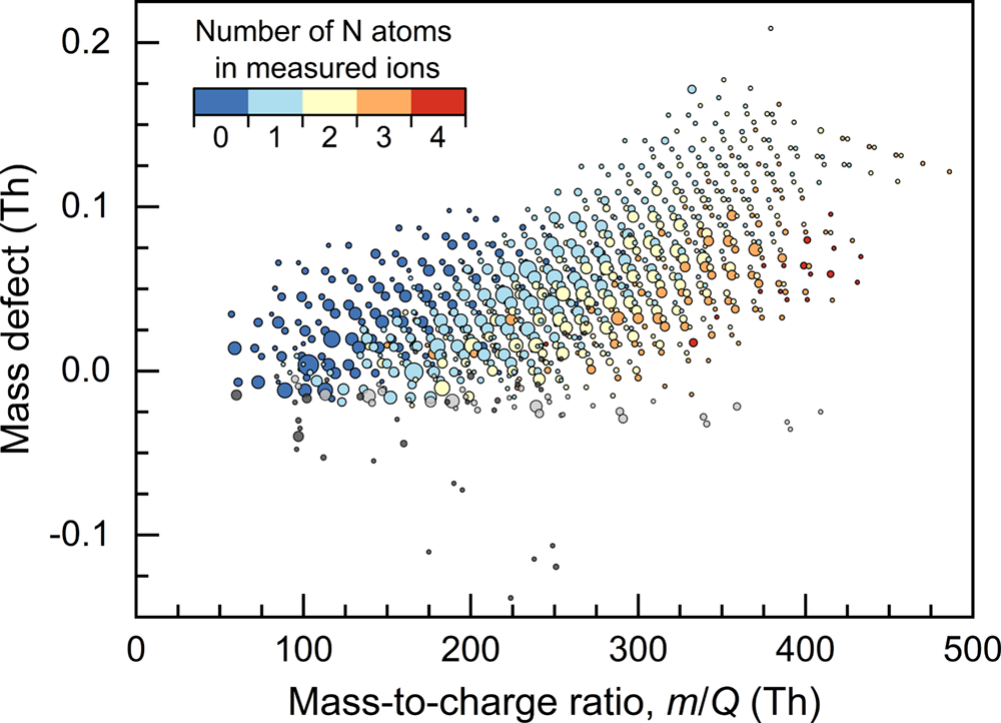
Gaseous compounds measured with chemical ionization Orbitrap mass spectrometry in the atmosphere of the city of Helsinki. The mass defect on the vertical axis is the difference between the exact and nominal mass-to-charge ratios of an ion. Only the peaks with assigned molecular formulas are shown. Compounds containing C, H, O, and possibly N are color-coded by the number of N atoms in each measured ion. Note that the reagent ion (NO_3_^–^) is not subtracted from the measured NO_3_^–^ clustered ions. Compounds containing F are shown in light gray. They did not have significant diurnal variations and were most probably from the sampling line. Other compounds are shown in dark gray. The sizes of markers indicate the concentrations of the measured compounds after the sensitivity correction.

The CI-Orbitrap with optimized parameters was used to measure atmospheric OOMs in the city of Helsinki. As shown in Figure 5, we detected a total of 935 ions and retrieved their molecular formulas. Most of the detected ions were OOMs, and a large fraction of those OOMs were nitrogen-containing OOMs due to the reactions between RO_2_ radicals and NO_x_ in the urban atmosphere.^34^

In addition to validating the feasibility to use the CI-Orbitrap for atmospheric measurements of trace OOMs, we have also tested parameters governing the sensitivity. In accordance with the chamber experiments, the signal of trace OOMs measured in the ambient increased as the microscan number increased (Figure S8). As shown in Figure 4a, the sensitivity of the CI-Orbitrap for the atmospheric measurements was very consistent with that of the chamber experiments, with the AGC target and the microscan number as the governing parameters. In addition, a relatively good consistency was observed between this study and the previous reports^15^ using a different CI-Orbitrap but the same AGC target and the same microscan number.

The measured atmospheric OOM signals emphasize the necessity to improve the sensitivity of the CI-Orbitrap as discussed above. The intensity distribution of measured OOMs in the city of Helsinki is given in Figure 4b. With AGC target = 10^5^ and microscan number = 1, only 12% (129 ions) of the measured peaks were above the 50% sensitivity threshold. In contrast, by improving the sensitivity with AGC target = 10^6^ and microscan number = 100, the fraction of the peaks above the 50% sensitivity threshold was increased to 58% (644 ions).

## Conclusions

We have shown that the CI-Orbitrap Fourier transform mass spectrometer can measure gaseous compounds with low concentrations in the atmosphere. To achieve this, we have investigated and then optimized the parameters governing the sensitivity of a Q Exactive Plus Orbitrap MS with chamber experiments and atmospheric measurements.

The sensitivity of the Orbitrap mass spectrometer decreases with a decreasing concentration of the measured compounds. We find that the sensitivity is positively correlated to the signal-to-noise ratio of individual spectra, which can be substantially improved by increasing the AGC target and the number of microscans. However, ultrahigh AGC targets (e.g., 5 × 10^6^) are not favorable because of the ion losses and/or fragmentation during the accumulation in the C-trap. Spectral averaging is also important for detecting trace compounds. The intensities of noise outliers in the averaged spectra are inversely proportional to the averaging. Although decreasing LOD by spectral averaging does not improve the sensitivity, it is beneficial to the identification of signal peaks among noise peaks. Other parameters influencing the measured signals, namely the eFT algorithm, the temperature of the inlet capillary, the RF of the S-Lens, and the range of mass-to-charge ratio, were also optimized.

By increasing the AGC target from 10^5^ to 10^6^ and the microscan number from 1 to 100, we decreased the 50% sensitivity threshold of a CI-Orbitrap by a factor of 50. Correspondingly, the number of measured compounds in the atmosphere of the city of Helsinki with signals above the 50% sensitivity threshold was increased from 129 to 644. The improved 50% sensitivity threshold corresponds to an estimated concentration of below 10^5^ molecules cm^–3^.

## Supporting Information Available

It includes the influence of the microscan number on reagent ion signal (Figure S1), The sensitivity of Orbitrap mass spectra with different averaging time (Figure S2), the influence of the enhanced Fourier transform algorithm on noise and the sensitivity (Figure S3), the influence of the inlet capillary temperature on signal (Figure S4), the influence of the S-lens radio frequency level on signal (Figure S5), the influence of the range of the mass-to-charge ratio of measured compounds on signal (Figure S6), and time series of measured ambient compounds with microscan numbers alternated every half an hour (Figure S7) (PDF)

## Abbreviations

AGC: automatic gain control
CI: chemical ionization
eFT: enhanced Fourier transform
FT: Fourier transform
LOD: limit of detection
MS: mass spectrometry
OOM: oxygenated organic molecule
RF: radio frequency
SNR: signal-to-noise ratio
ToF: time-of-flight
VOC: volatile organic compound
