# Supplementary material for: Improving the Sensitivity of Fourier Transform Mass Spectrometer (Orbitrap) for Online Measurements of Atmospheric Vapors
Source: Anal Chem. 2022 Nov 7;94(45):15746–53. doi: 10.1021/acs.analchem.2c03403 (PMC9670027; doi:10.1021/acs.analchem.2c03403)
Supplement: Supplementary file 1 — ac2c03403_si_001.pdf [file ac2c03403_si_001.pdf]

Supplementary Information for

Improving the sensitivity of Fourier transform mass spectrometer (Orbitrap) for online measurements of atmospheric vapors

Runlong Cai[†,*], Wei Huang[†], Melissa Meder[†], Frederic Bourgain[‡], Konstantin Aizikov[§], Matthieu Riva[‡], Federico Bianchi[†], Mikael Ehn[†,*]

[†]Institute for Atmospheric and Earth System Research/Physics, Faculty of Science, University of Helsinki, 00014 Helsinki, Finland

[‡]Univ Lyon, Université Claude Bernard Lyon 1, CNRS, IRCELYON, 69626, Villeurbanne, France

[§]Thermo Fisher Scientific (Bremen), 28199 Bremen, Germany

[*]Correspondence to Runlong Cai, runlong.cai@helsinki.fi and Mikael Ehn, mikael.ehn@helsinki.fi

Table of Contents

Figure S1........ 2

Figure S2........ 2

Figure S3........ 3

Figure S4........ 3

Figure S5........ 4

Figure S6........ 4

Figure S7........ 5

Figure S8........ 5

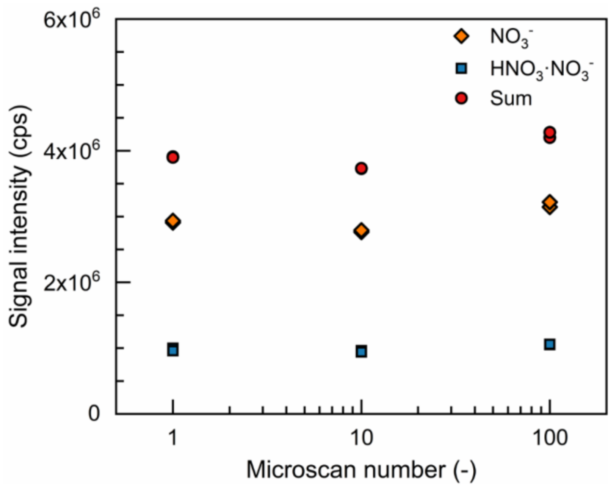

Figure S1. The influence of the number of averaged microscans (time-domain transients) on the measured reagent ions (NO3- and HNO3·NO3-) during the chamber experiments. This figure shows the stability of the chemical ionization inlet during the tests with a varying microscan number. It also indicates that the microscan number negligibly affects the measured signal of compounds with high concentrations.

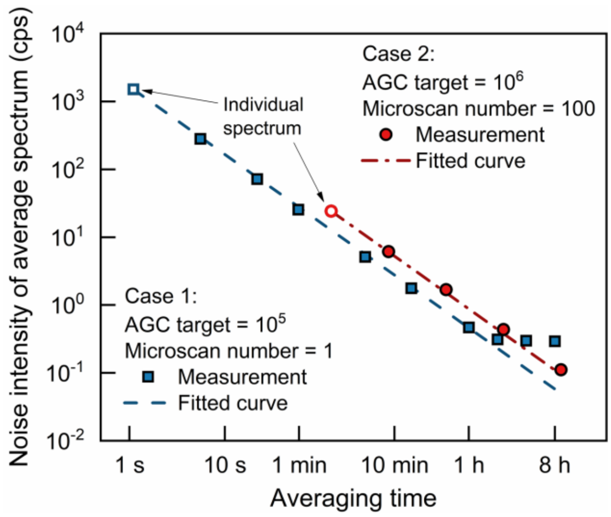

Figure S2. The intensity of noise peak of the averaged spectrum as a function of the averaging time. Noise peaks in a spectrum are outliers of the noise above a certain threshold, hence spectral averaging does not affect noise but it can reduce the height of outlier peaks. The data with averaging time longer than 1 h for case 1 were not used for fitting. The open markers indicate the spectra with microscan number = 1, *i.e.*, individual spectra.

For certain settings of the Orbitrap parameters, there is a saturation point of the noise peak intensity for spectral averaging. This figure shows that with AGC target = 10^5 and microscan number = 1, increasing the averaging time from 2 h to 8 h no longer reduces the noise peak intensity as they significantly interfere with each other; that is exactly where increasing the AGC target and the number of microscans will further decrease the noise and improve the detection.

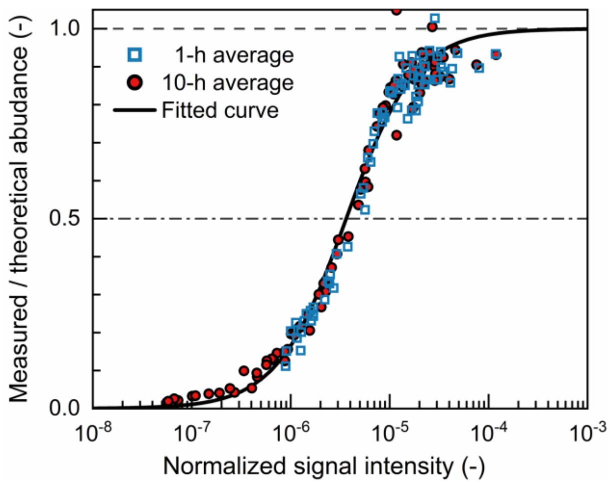

Figure S3. The sensitivity of Orbitrap mass spectra with different averaging time. The data were measured in the chamber experiments with automatic gain control target = 10^6 and micro scan number = 100. The horizontal axis is the signal intensity normalized by dividing it by the total intensity of the reagent ions ($[NO_3^-]$ + $[HNO_3{\cdot}NO_3^-]$). The vertical axis is the measured abundance of ions containing less abundant isotopes divided by the theoretical abundance calculated from the primary peak. Only the isotope peaks are shown in this figure.

This figure shows that spectral averaging decreases the limit of detection though it does not affect the sensitivity. As a result, signals with normalized intensity lower than 10^{-6} could be identified by extending the averaging time from 1 h to 10 h.

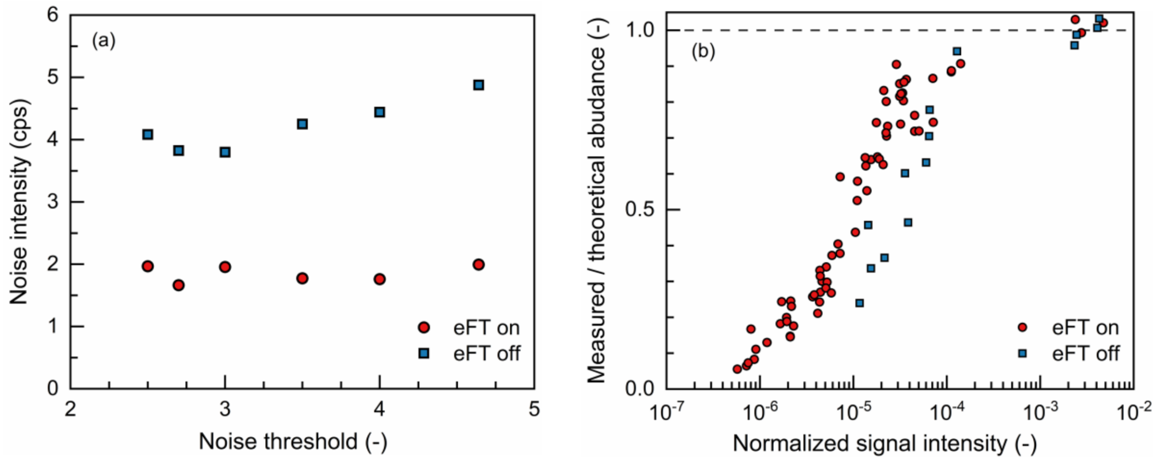

Figure S4. The influences of the enhanced Fourier transform (eFT) algorithm on (a) the noise in averaged spectra and (b) the sensitivity of chemical ionization Orbitrap mass spectrometer.

The data were measured in the chamber experiments with automatic gain control target = 10^6, micro scan number = 100, and averaging time = 1 h. The noise threshold in panel (a) is a tunable parameter of the Orbitrap determining the assumed signal-to-noise ratio for a single spectrum[1]. The normalized signal intensity in (b) is the ratio of the measured intensity to the total intensity of the reagent ions ($[NO_3^-]$ + $[HNO_3{\cdot}NO_3^-]$). The vertical axis in (b) shows the measured isotopic abundance of an ion divided by its theoretical abundance (See Figure 4 in the main text).

The eFT uses the interpolation result between the absorption and magnitude FT results instead of the magnitude FT result only[2]. The noise of absorption FT is lower than that of magnitude FT[3]. Hence, eFT reduces noise (panel a) and improves the sensitivity (panel b). The noise threshold in Figure S4a was not found to influence the sensitivity significantly in this study.

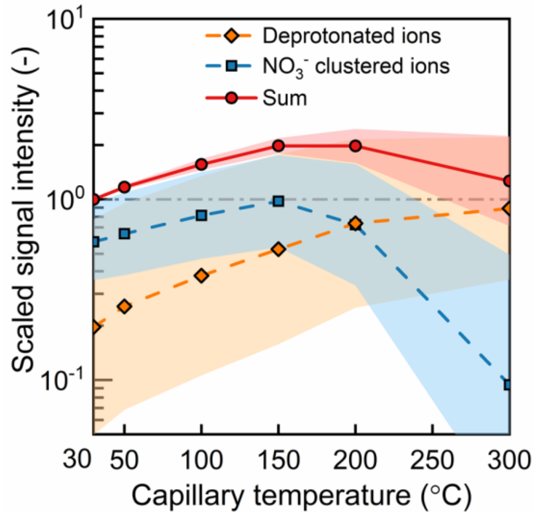

Figure S5. The influences of the capillary temperature on the measured oxygenated organic molecules during the chamber experiments. Only the compounds for which the deprotonated and NO_3^- clustered ions are both detected are shown in this figure. The signal intensities are scaled by dividing them by their corresponding sum at the lowest capillary temperature. The lines and markers show the geometric means of the signal intensities of different compounds, and the shaded areas indicate the geometric standard deviations.

The inlet capillary of the Orbitrap was heated to reduce the losses of measured compounds to the capillary wall. This figure shows that when the capillary temperature was increased from 30 °C to 150 °C, the measured signal was increased by an average factor of 2. However, the measured signal decreased when the capillary temperature was higher than 150 °C, especially for NO_3^- clustered ions. The signal of deprotonated ions increased monotonically with the capillary temperature in the test conditions because the decomposition of a NO_3^- clustered ion might produce a corresponding deprotonated ion.

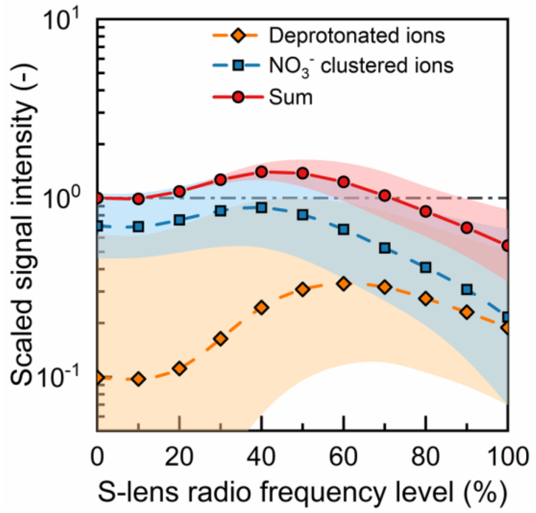

Figure S6. The influences of the S-lens radio frequency (RF) level on the measured oxygenated organic molecules during the chamber experiments. Only the compounds for which the deprotonated and NO_3^- clustered ions are both detected are shown in this figure. The signal intensities are scaled by dividing them by their corresponding sum at the lowest RF level. The lines and markers show the geometric means of the signal intensities of different compounds, and the shaded areas indicate the geometric standard deviations.

The S-lens RF level affects the transmission of ions and the RF electric field may also cause the fragmentation of ions. The NO_3^- clustered ions start to decrease at a lower RF level compared to the deprotonated ions because the fragmentation of a NO_3^- clustered ion may produce a corresponding deprotonated ion. This also indicates a significant cluster fragmentation at high RF levels. The optimal S-lens RF level corresponding to the maximum signal for the chamber experiments in this study was found to be 40, which is close to the values reported in previous studies for the Orbitrap[4, 5].

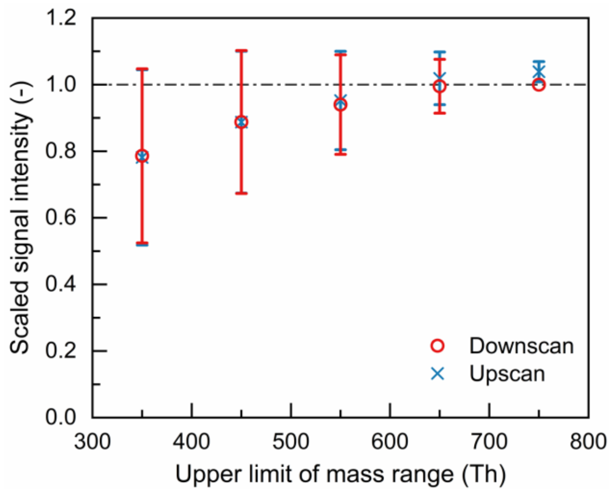

Figure S7. The influence of the range of mass-to-charge ratio (mass range for short) on the measured OOMs during the chamber experiments. The lower limit of the mass range was fixed at 50 Th. The upper limit of the mass range was decreased from 750 Th to 300 Th (downscan) and then was increased back (upscan). The signal intensities are scaled by dividing them by their corresponding value at 750 Th in the down scan. The marker and variation bar indicate the mean and standard deviation, respectively, of the signal intensities for different ions. This figure shows an unexpected weak dependency of the measured signal on the mass range of the spectrum.

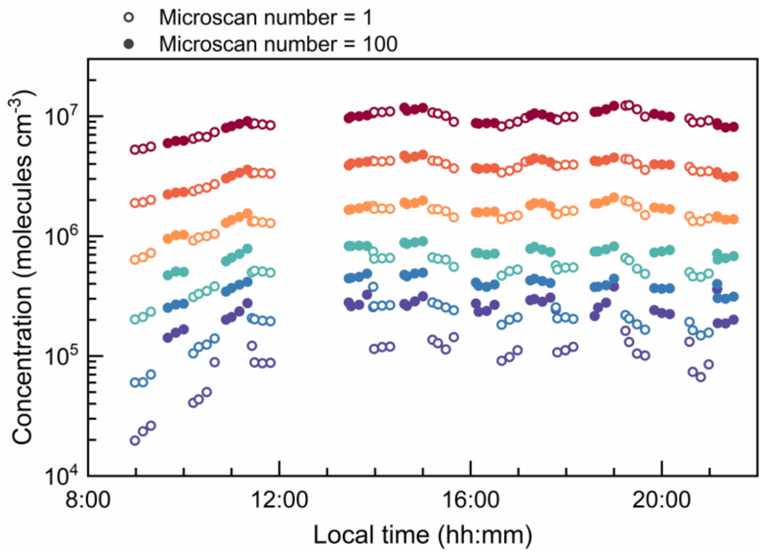

Figure S8. The time series of measured gaseous compounds in the atmosphere of the city of Helsinki with a nitrate chemical ionization Orbitrap. The microscan number was alternated between 1 and 100 every half an hour. The raw concentration was estimated by assuming a calibration factor of 1×10^{10} cm^{-3} and the non-linear sensitivity of Orbitrap was not corrected for the data shown in this figure. The measured compounds are grouped according to their intensities and the markers show the mean value of each group. Compounds with concentrations higher than 10^7 cm^{-3} at 9 am are not shown. This figure shows that using a high microscan number improves the sensitivity of the Orbitrap to compounds with low concentrations, which is consistent with Figure 3 in the main text.

REFERENCES

1. Makarov, A.; Denisov, E., Dynamics of ions of intact proteins in the Orbitrap mass analyzer. *J. Am. Soc. Mass Spectrom.* **2009,** *20* (8), 1486-95.

2. Lange, O.; Damoc, E.; Wieghaus, A.; Makarov, A., Enhanced Fourier transform for Orbitrap mass spectrometry. *Int. J. Mass Spectrom.* **2014,** *369*, 16-22.

3. Qi, Y.; Barrow, M. P.; Li, H.; Meier, J. E.; Van Orden, S. L.; Thompson, C. J.; O'Connor, P. B., Absorption-mode: the next generation of Fourier transform mass spectra. *Anal. Chem.* **2012,** *84* (6), 2923-9.

4. Scheltema, R. A.; Hauschild, J. P.; Lange, O.; Hornburg, D.; Denisov, E.; Damoc, E.; Kuehn, A.; Makarov, A.; Mann, M., The Q Exactive HF, a Benchtop mass spectrometer with a pre-filter, high-performance quadrupole and an ultra-high-field Orbitrap analyzer. *Mol. Cell. Proteom.* **2014,** *13* (12), 3698-708.

5. Criscuolo, A.; Zeller, M.; Fedorova, M., Evaluation of Lipid In-Source Fragmentation on Different Orbitrap-based Mass Spectrometers. *J. Am. Soc. Mass Spectrom.* **2020,** *31* (2), 463-466.
